# Oxygen‐Rich Lithium Oxide Phases Formed at High Pressure for Potential Lithium–Air Battery Electrode

**DOI:** 10.1002/advs.201600453

**Published:** 2017-05-19

**Authors:** Wenge Yang, Duck Young Kim, Liuxiang Yang, Nana Li, Lingyun Tang, Khalil Amine, Ho‐Kwang Mao

**Affiliations:** ^1^ Center for High Pressure Science and Technology Advanced Research (HPSTAR) Shanghai 201203 China; ^2^ High Pressure Synergetic Consortium (HPSynC) Geophysical Laboratory Carnegie Institution of Washington 9700 S Cass Avenue Argonne IL 60439 USA; ^3^ Chemical Science & Engineering Division Argonne National Laboratory 9700 S Cass Avenue Argonne IL 60439 USA

**Keywords:** high pressure, lithium–air batteries, phase transition, redox procedure, superoxides

## Abstract

The lithium–air battery has great potential of achieving specific energy density comparable to that of gasoline. Several lithium oxide phases involved in the charge–discharge process greatly affect the overall performance of lithium–air batteries. One of the key issues is linked to the environmental oxygen‐rich conditions during battery cycling. Here, the theoretical prediction and experimental confirmation of new stable oxygen‐rich lithium oxides under high pressure conditions are reported. Three new high pressure oxide phases that form at high temperature and pressure are identified: Li_2_O_3_, LiO_2_, and LiO_4_. The LiO_2_ and LiO_4_ consist of a lithium layer sandwiched by an oxygen ring structure inherited from high pressure ε‐O_8_ phase, while Li_2_O_3_ inherits the local arrangements from ambient LiO_2_ and Li_2_O_2_ phases. These novel lithium oxides beyond the ambient Li_2_O, Li_2_O_2_, and LiO_2_ phases show great potential in improving battery design and performance in large battery applications under extreme conditions.

## 1 Introduction

Lithium‐ion batteries can store a large amount of energy in a relatively small volume and have been extensively used as rechargeable batteries for portable electronics. Over the last decade, lithium–air (Li–O_2_) batteries have attracted great attention on new battery development as they have a gravimetric energy density close to that of gasoline.^1^, ^2^, ^3^, ^4^ Li–O_2_ batteries use the oxidation of lithium at the anode and the reduction of oxygen at the cathode to create a current. It is commonly known that the Li–O_2_ battery often forms insoluble discharge products, mainly oxides, which accelerate the degradation of the electrode and electrolyte, thus reducing the cycle stability and efficiency.^5^, ^6^, ^7^, ^8^, ^9^, ^10^, ^11^ Recently, novel approaches to combat this degradation have been actively pursued by forming LiOH^12^ or stable LiO_2_.^13^ By analogy to sodium^14^ and potassium^15^ superoxide, lithium superoxide has been considered as an alternative cathode based on reduced graphene oxide.^13^ The major problem is that the lithium superoxide is not stable at ambient condition. Investigation of the stability of these known phases and new forms of lithium oxides would greatly extend our knowledge on improving the capability and lifetime of lithium–air batteries.

There have been some studies on the lithium oxides at various conditions. Yao et al. investigated the thermal stabilities of Li_2_O_2_ and Li_2_O at the battery working temperature.^5^ Shi et al. reported density functional theory (DFT) calculations and experimental confirmation of Li_3_O_4_ nanoparticles as a discharge byproduct.^6^ Also, the recent discovery of lithium superoxide as a discharge product has attracted much attention, and it is considered as a highly promising candidate for the next‐generation lithium battery.^7^, ^8^, ^9^, ^10^, ^11^, ^13^

Pressure as an efficient way to tune the lattice and electronic structure has been extensively used to explore novel material synthesis. Sodium chloride displays the polymorphisms Na_3_Cl and NaCl_3_ under pressure.^16^ The newly discovered stable phases magnesium and iron peroxides (MgO_2_ and FeO_2_) formed from a high pressure and temperature redox reaction provide a new look at oxygen and hydrogen cycling in the deep earth interior.^17^, ^18^ Ultrahigh pressure sulfur hydride demonstrates superconducting behavior at record high temperature, 203 K.^19^ We addressed two questions. What is the stability of the aforementioned lithium oxides under high pressure? Can we synthesize even higher oxygen content lithium oxides by application of pressure? In particular, we investigated the structural stability of pure Li_2_O_2_ and mixture of Li_2_O_2_ with oxygen at high pressure, and found that the pure Li_2_O_2_ is robustly stable up to 57 GPa, while the mixture of Li_2_O_2_ with adequate oxygen environment produces a series of oxygen‐rich phases under high pressure and temperature. We also applied DFT calculations to search the possible thermodynamically stable phases, and found that the calculated results matched the experimental observations. This may inspire broader applications of these new forms of lithium oxides.

## 2 Results and Discussion

**Figure 1:**
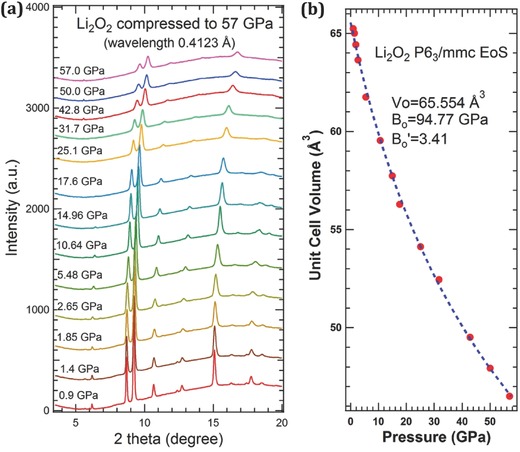
High‐pressure XRD measurements of Li_2_O_2_ up to 57 GPa show the robustly stable phase P6_3_/mmc at room temperature. a) The XRD profiles at various pressures up to 57 GPa. b) Unit cell volume versus pressure. The dashed line presents the fitting result of the third‐order Birch–Murnaghan equation of state with bulk modulus *B*_o_ = 94.77 GPa and its derivative with respect to pressure *B*_o_′ = 3.41.

At ambient condition, both lithium oxide (Li_2_O) and lithium peroxide (Li_2_O_2_) are thermodynamically stable. During the charge–discharge cycling of Li–O_2_ batteries, Li_2_O_2_ plays the major role. It has been reported that a small amount of lithium superoxide (LiO_2_) nanoparticles can form on the surface of the Li_2_O_2_.^7^, ^8^, ^9^, ^13^ To check the stability and possible formation of high‐pressure oxygen‐rich lithium oxides, we started with pure Li_2_O_2_ (Sigma‐Aldrich) powder loaded in a diamond anvil cell without any pressure‐transmitting medium for a test at high pressure and room temperature. The Li_2_O_2_ powder was compressed to 57 GPa and subjected to synchrotron X‐ray diffraction (XRD) to follow the structural evolution as a function of pressure. **Figure** **1**a shows the angle‐dispersive XRD profiles for the powder at various pressures. All diffraction peaks shift to higher 2θ as pressure is increased. Up to the highest pressure measured, no symmetry change was observed. The XRD patterns can be described by the ambient P6_3_/mmc symmetry to the highest pressure. As shown in Figure 1b, the compression curve (unit cell volume vs pressure) can be fit to the third‐order Birch–Murnaghan equation of state with bulk modulus *B*_o_ = 94.77 GPa and derivative of *B*_o_ as 3.41.

On the second run, Li_2_O_2_ powder was loaded with cryogenically condensed liquid oxygen in the sample chamber of a diamond anvil cell. The mixture of Li_2_O_2_ powder and liquid oxygen was precompressed to 15.0 GPa, followed by heating to 2200 K at high pressure using a Nd:YAG laser system. After the laser heating, the pressure in the sample chamber dropped to 14.4 GPa. As the Li_2_O_2_ powder is semitransparent and no thermal shielding layers were used to prevent heat from conducting away from the diamond anvils, a high laser power (total 80 W from a double‐side laser heating unit) was needed to heat the sample to the maximum temperature of 2200 K. A 2D raster scan was performed around the laser‐processed center of the sample, and the powder diffraction pattern was collected at each scanning location. We clearly saw totally different diffraction profiles in the laser‐processed center area, with the most diffraction peaks being spotty, indicating new structures had been synthesized in the form of many small single‐crystal grains. In the diffraction profile of the central 20 µm region where the laser heating was applied, one broad smooth powder ring appeared near 2θ = 7.8°, which was not evident outside this region. Upon opening the diamond anvil cell, we realized that the central area of the anvil had been damaged by the high‐power laser beams (see Figure S1 in the Supporting Information), and part of the carbon had been removed from the diamond culet surface.

On the third run, the Li_2_O_2_ powder was sandwiched between two thin layers of LiF pellets, and loaded with liquid oxygen in a diamond anvil cell. The sample was compressed to 50.0 GPa, followed by laser heating. The pressure inside the chamber dropped to 48.0 GPa after cooling to room temperature. With the thermal insulator LiF layers, only 20 W power was needed to heat the sample above 2000 K, and no damage was observed on the diamond surfaces. Synchrotron X‐ray diffraction was taken at the laser‐processed location. The diffraction profile changed dramatically from the previous two runs, indicating that new structures had been synthesized.

To clarify the high temperature requirement for synthesizing these new phases from previous three runs, we conducted one more room temperature compression experiment in a diamond anvil cell. Mixture of Li_2_O_2_ powder and oxygen was compressed to 53 GPa without laser heating. The selected XRD patterns around 15 and 50 GPa are shown in Figure S2 (Supporting Information). All diffraction peaks can be characterized by the known ambient pressure Li_2_O_2_ phase (P6_3_/mmc) and oxygen ε‐O_8_ phase (C2/m, a stable high pressure phase with pressure greater than 10 GPa) up to the highest pressure in this run.

**Figure 2:**
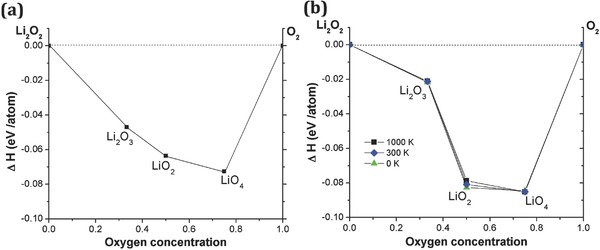
The predicted convex hulls of Li–O at a) 14.4 GPa and b) 48 GPa. To check the thermostability, the temperature effect has been considered from 0 to 1000 K. At 48 GPa, the phonon stability of LiO_2_ (P4/mbm) decreases as the temperature increases, but no temperature effects are observed for Li_2_O_3_ (Im‐3m) or LiO_4_ (Ibam) phases.

Crystal structure searching based on DFT was conducted to understand the energy landscape of the Li–O compounds in the experimental environments. With two end members, Li_2_O_2_ and O_2_, we predicted possible stoichiometry and crystal structure at 15 and 48 GPa, as shown in **Figure** **2**. At 15 GPa, we found that Li_2_O_3_, LiO_2_, and LiO_4_ are stable phases, with LiO_2_ and LiO_4_ remaining stable even at 48 GPa. At 15 GPa, Li_2_O_3_ and LiO_4_ have Im‐3m and Ibam space groups, respectively. It is interesting to see that for LiO_2_, the transition pressure from the known ambient Pnnm phase to the new P4/mbm phase is at 12 GPa, close to our second run experimental condition around 15 GPa. The enthalpy plot of LiO_2_ is shown in Figure S3 (Supporting Information). The formation enthalpy of stable phases with respect to decomposition into Li_2_O_2_ and O_2_ is less than 0.1 eV per atom, and some phases possess total energy close to that of the stable phases. Thus, it is likely that the temperature effect could play a role in the thermodynamic stability.

To understand the thermodynamic stability of each phase, we conducted phonon calculations. Clearly, these candidate phases show stable phonon dispersion relations (see Figure S4 in the Supporting Information). To compare the synthesizing condition with high temperature, we also tested the temperature effect up to 1000 K on the formation enthalpy at 48 GPa, using a quasiharmonic approximation. While other phases remain almost constant, LiO_2_ shows a tendency to become less stable at higher temperature.

Bader charge analysis provided further insight into the bonding nature between Li and O_2_ units. The effective charges on the Li/O_2_ unit in Li_2_O_2_ are +1.6 and −1.6, and the O—O bonding distance is 1.50 Å, compared to 1.21 Å in the solid oxygen. By taking more oxygen, the effective charge of the O_2_ unit is reduced to −1.07 (Li_2_O_3_), −0.82 (LiO_2_), and −0.41 (LiO_4_), forming superoxides. The corresponding bond length of O_2_ changes to 1.36, 1.31, and 1.25 Å, respectively, which are close to that of solid oxygen. The effective charge of Li remains almost constant and occupies similar volumes in all Li–O compounds. Therefore, we speculate that Li–O compounds under pressure take advantage of having smaller molecular volume of oxygen units by reducing electrons at antibonding states. In other words, Li–O compounds tend to form superoxides under pressure to lower the total energy by reducing their O_2_ volume. This trend can be also observed in the phonon dispersions curves (see Figure S4 in the Supporting Information). The calculated phonon dispersion of Li_2_O_2_ at 48 GPa was stable, a finding in good agreement with our pure Li_2_O_2_ high pressure experiments. Increasing oxygen concentrations in oxygen‐rich phases show higher vibrational modes of O_2_ units, and we can see that the O_2_ phonon band splits from the lower energy phonon bands.

**Figure 3:**
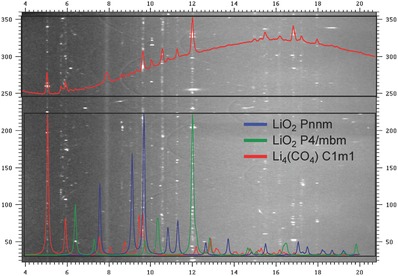
High‐pressure XRD pattern after laser heating at 14.4 GPa. The cake view of the 2D diffraction pattern shows many sharp diffraction spots after laser heating, indicating new phases are present in the form of many small single crystals. The total integrated intensity profile (upper inset) can be well described by LiO_2_ Pnnm, LiO_2_ P4/mbm, and Li_4_(CO_4_) C1m1 phases (lower inset). The Reitveld refinement of the XRD profile with the three candidate structures are shown in Figure S5 (Supporting Information).

For interpreting the 14.4 GPa XRD results, we considered that the carbon that escaped from the diamond anvil surface may participate in the new compound formation. The powder diffraction profile fit well with the Li_4_CO_4_ C1m1 phase predicted by Cancarevic et al.,^20^ as well as the LiO_2_ (Pnnm) and LiO_2_ (P4/mbm) phases from our DFT calculations. **Figure** **3** shows the unrolled XRD pattern at 14.4 GPa and the simulated diffractions from Li_4_CO_4_ (C1m1), LiO_2_ (Pnnm), and LiO_2_ (P4/mbm) phases. The distinguishing feature on the diffraction pattern is the very spotty diffraction rings, indicating micrometer‐sized grains with well‐annealed single crystals. The high temperature promotes the chemical reaction between Li_2_O_2_ and oxygen, and also allows the crystal growth of the new phases. The Bragg diffraction positions from these three phases match the *d*‐spacings from the powder diffraction. We conducted Reitveld refinement on the integrated 1D pattern with the above three phases. Although the intensity cannot be fitted very well due to the spotty patterns, the lattice parameters can be obtained with this refinement. Roughly, each phase occupies about 1/3 volumetrically. The fitting result and parameters of these three phases are displayed in Figure S5 and Table S1 (Supporting Information), respectively. With 14.4 GPa pressure and high temperature, the data indicate that the following redox reactions take place

(1) $$2{\mathrm{Li}}_{2}O_{2}\;+\;C\;\to \;{\mathrm{Li}}_{4}{\mathrm{CO}}_{4}$$

(2) $${\mathrm{Li}}_{2}O_{2}\;+\;O_{2}\;\to \;2{\mathrm{LiO}}_{2}$$

Thus, the new compounds Li_4_CO_4_ and LiO_2_ are formed with further reduction and oxidation from pristine Li_2_O_2_ by carbon and oxygen, respectively. Both Pnnm and P4/mbm LiO_2_ phases are present at this pressure, which is close to the DFT calculation result on the Pnnm→P4/mbm transition pressure near 12 GPa. Cancarevic et al. proposed that the Li_4_CO_4_ (C1m1) phase could be synthesized in the pressure range 80–110 GPa.^21^ Grzechnik et al. attempted to form Li_4_CO_4_ from a mixture of Li_2_O and Li_2_CO_3_ but failed to obtain the predicted Li_4_CO_4_ phase up to 25 GPa and 1100 K.^20^ Our diamond anvil test results show the successful synthesis of Li_4_CO_4_ from Li_2_O_2_ and vapor carbon at 14.4 GPa and 2200 K via a redox reaction, which provides a much lower pressure route to form lithium orthocarbonate. For comparison, we also took diffraction patterns at off‐center locations. In the bottom panel of Figure S5 (Supporting Information), the XRD profile taken at 30 µm away from the laser center is shown, where much lower heating temperature is expected. Very small amount of Li_4_CO_4_ was formed and some not well formed LiO_2_ P4/mbm and Pnnm phases (broad and low intensity peaks) were present, which indicates the temperature is an important parameter for the new LiO_2_ phase synthesis.

**Figure 4:**
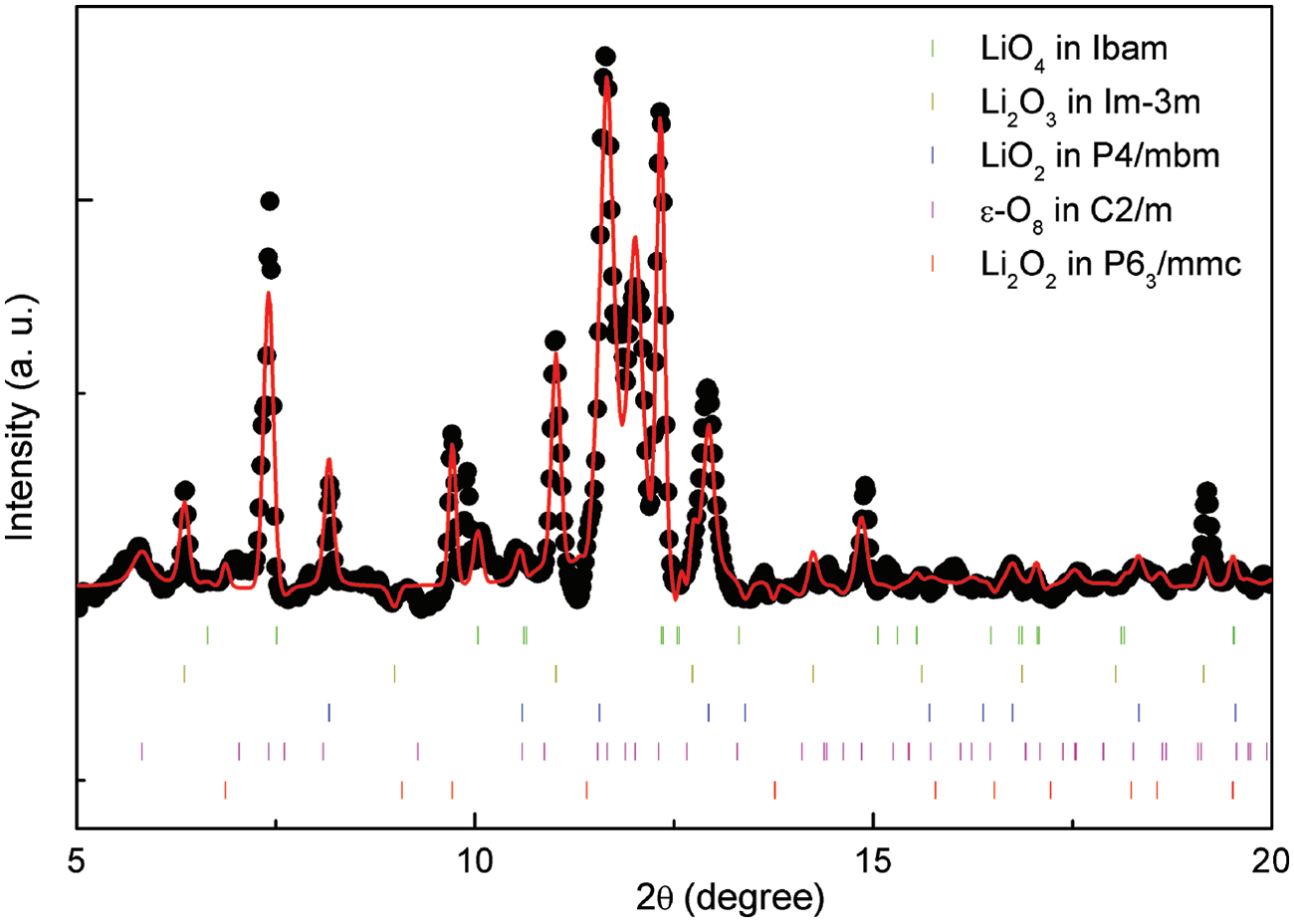
High pressure XRD pattern after laser heating at 48 GPa and the corresponding Reitveld refinement with four high pressure oxides and ε‐O_8_ phases. The black dotted and continuous red curves are experimental and refined diffraction patterns. The color vertical bars are the Bragg positions of four oxides and ε‐O_8_ phase at 48 GPa. New oxygen‐rich phases LiO_4_ Ibam (24.99%), Li_2_O_3_ Im‐3m (36.82%), and LiO_2_ P4/mbm (3.27%) are present along with ε‐O_8_ C2/m (13.70%) and Li_2_O_2_ P6_3_/mmc (21.22%) phases.

Because at 48 GPa, the XRD Debye–Scherrer rings are smooth, we obtained good powder diffraction patterns for structural Reitveld refinement. As presented in **Figure** **4**, the best fit of the XRD pattern is from the combination of phases LiO_2_ (P4/mbm), LiO_4_ (Ibam), and Li_2_O_3_ (Im‐3m) along with high pressure ε‐O_8_ phase (C2/m)^22^ and pristine Li_2_O_2_ phase (P6_3_/mmc). From the fitting volumetric percentage, the amount of LiO_2_ is quite small (3.27%), but the amount of Li_2_O_2_ has increased dramatically. The following chemical reactions can be considered

(3) $${\mathrm{Li}}_{2}O_{2}\;+\;O_{2}\;\to \;2{\mathrm{LiO}}_{2}$$

(4) $$2{\mathrm{Li}}_{2}O_{2}\;+\;O_{2}\;\to \;2{\mathrm{Li}}_{2}O_{3}\;\mathrm{and/or}\;4{\mathrm{LiO}}_{2}\;\to \;2{\mathrm{Li}}_{2}O_{3}\;+\;O_{2}$$

(5) $${\mathrm{Li}}_{2}O_{2}\;+\;3O_{2}\;\to \;2{\mathrm{LiO}}_{4}\;\mathrm{and/or}\;{\mathrm{LiO}}_{2}\;+\;O_{2}\;\to \;{\mathrm{LiO}}_{4}$$

(6) $$3{\mathrm{LiO}}_{2}\;\to \;{\mathrm{Li}}_{2}O_{2}\;+\;{\mathrm{LiO}}_{4}\;\mathrm{and/or}\;2{\mathrm{LiO}}_{2}\;\to \;{\mathrm{Li}}_{2}O_{2}\;+\;O_{2}$$

In the above redox reactions, the LiO_2_ is mainly an intermediate product and largely converted to Li_2_O_3_ and LiO_4_ phases. At this high pressure, only the P4/mbm phase of LiO_2_ is observed, and the Li_2_O_2_ has the same structure as that at ambient pressure, consistent with the conclusion from run 1. Because of the thermal insulator layers of LiF, the diamond anvils survived, and no carbon was involved in the redox reaction at 48 GPa. In the above redox reactions, the LiO_2_ takes a new form as P4/mbm structure, whereas the LiO_4_ stays at Ibam phase. Additionally, the stable Li_2_O_3_ phase can be successfully detected from the powder diffraction. The strong peak at 2θ = 7° was attributed to the ε‐O_8_ phase.^22^ Based on DFT calculations, although the LiO_2_ is a thermally stable phase under high temperature and pressure, it is largely combined with Li_2_O_2_ and O_2_ to form Li_2_O_3_ and LiO_4_, respectively, and LiO_2_ itself becomes less stable at high temperature and may decompose into Li_2_O_2_ and O_2_, or Li_2_O_2_ and LiO_4_. This decomposition might lead to only a small percentage of LiO_2_ at 48 GPa (only 3.27%).

Comparing the DFT calculation and experimental results, we notice the discrepancy of Li_2_O_3_ phase in different pressures. Although the Li_2_O_3_ is predicted as a stable phase starting at 15 GPa by DFT calculation, we did not observe this structure with XRD; but at 48 GPa, more than 1/3 of the diffraction intensity was attributed to Li_2_O_3_. The DFT calculations did not indicate this phase at this pressure, as shown by the convex hulls in Figure 2. This discrepancy between DFT calculation and experimental observation could be due to the high kinetic energy of the Li_2_O_3_ phase. At 14.4 GPa, the pressure and temperature are not enough to overcome the kinetic barrier to form Li_2_O_3_ phase, although thermodynamically it is stable; at 48 GPa, however, the pressure and temperature bring Li_2_O_3_ to a metastable phase, as we observed experimentally despite the DFT calculation not favoring formation of this phase at this pressure. Authors noticed a similar DFT calculation work was posted on arXiv, where some lithium‐rich lithium oxides are predicted as well.^23^

## 3 Conclusion

**Figure 5:**
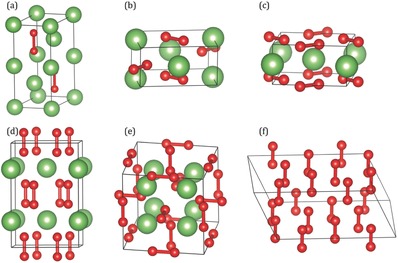
Crystal structures of five lithium oxide and ε‐O_8_ phases. The atomic structure arrangements in unit cell: a) ambient pressure Li_2_O_2_ (P6_3_/mmc), b) ambient pressure LiO_2_ (Pnnm), c) high pressure LiO_2_ (P4/mbm), d) high pressure LiO_4_ (Ibam), e) high pressure Li_2_O_3_ (Im‐3m), f) high pressure ε‐O_8_ (C2/m) phase. Red and green spheres represent oxygen and lithium atoms, respectively.

In conclusion, under high pressure and temperature, several stable phases of oxygen‐rich lithium oxides can be synthesized (see **Figure** **5**). Combining the DFT predictions and in situ structural measurements at high pressure, we have successfully confirmed three new stable oxygen‐rich lithium oxide phases: LiO_2_ (P4/mbm), Li_2_O_3_ (Im‐3m), and LiO_4_ (Ibam). Also, the Li_2_O_2_ phase (P6_3_/mmc) is robustly stable up to 57 GPa at room temperature. The high pressure LiO_2_ and LiO_4_ phases can be considered as lithium atoms inserted between the layers of ε‐O_8_ phase, while the Li_2_O_3_ structure inherits the local atomic arrangements from the ambient Li_2_O_2_ and LiO_2_ (Pnnm) structures. At increasing temperature, the high‐pressure LiO_2_ (P4/mbm) phase losses its stability and converts to LiO_4_ and Li_2_O_3_ or decomposes to Li_2_O_2_ and O_2_. This variety of lithium oxides provides a rich field for discovering candidate electrode materials for lithium–oxygen batteries. For instance, the LiO_4_ phase could offer an opportunity of using the material as electrode for a close and novel lithium oxygen cell since LiO_4_ can provide the O_2_ needed in this case.

## 4 Experimental Section

*High Pressure XRD Measurement*: Synchrotron XRD measurements in the angle‐dispersive diffraction mode were conducted in a diamond anvil cell at beamlines 16BM‐D and 16ID‐B by the High Pressure Collaborative Access Team (HPCAT) at the Advanced Photon Source, Argonne National Laboratory. The monochromatic beam was focused to around 5–10 µm full width at half maximum, and the powder diffraction patterns were recorded by a Mar345 image plate at the 16BM‐D station and a Pilatus 1M detector at the 16ID‐B station. The 2D patterns were integrated to a 1D profile with Fit2d software, and the Rietveld structural refinements were conducted with the GSAS package.^24^

For the first run, the X‐ray wavelength was 0.4123 Å, and the pressure was calibrated by the ruby luminescence method. In the second run, a mixture of Li_2_O_2_ powder and liquid oxygen was compressed to 15 GPa, followed by high power laser heating to 2200 K. This heat treatment vaporized the carbon from the anvil surface, which did not have a thermal insulating layer, thus adding a small amount of carbon to the chemical reaction. The surface damage of the anvil after laser heating showed clear evidence of missing carbon from the anvil. The X‐ray wavelength used was 0.39 995 Å. On the third run, with the help of thermal insulator layers of LiF, no diamond anvil damage was observed after laser heating to above 2000 K. The X‐ray wavelength used was 0.40 663 Å. In both runs 2 and 3, no ruby was loaded in the diamond anvil cells to avoid the possible chemical reaction during laser heating. The pressures were calibrated with the diamond Raman signal. For the final comparison run on the mixture of Li_2_O_2_ and oxygen at room temperature up to 53 GPa, the X‐ray wavelength used was the same as third run. Since no heat treatment was involved, a small Ruby ball was loaded in the sample chamber as pressure calibrant.

*Ab Initio Crystal Structure Searching*: The first principle calculations were performed in the framework of density functional theory^25^, ^26^ through the package VASP.^27^ The generalized gradient approximation of Perdew, Burke, and Ernzerhof was implemented to describe the exchange correlation functions.^28^, ^29^ Pseudopotentials were used with 3 valence electrons for Li (1s^2^2s1) and 6 for O atoms (2s^2^2p4). For crystal structure searching, we used USPEX with a plane‐wave basis set cutoff energy of 800 eV.

*Phonon Dispersion Curves from First Principles*: Phonon calculations were conducted based on density functional perturbation theory^30^ implemented in VASP software in connection with phonopy software.^31^ It was found that phonon dispersions are stable for LiO_2_, Li_2_O_3_, and LiO_4_ at 14.4 and 48 GPa, as shown in Figure S4 (Supporting Information).

## Supporting information

Supplementary
