# Supplementary material for: Oxygen‐Rich Lithium Oxide Phases Formed at High Pressure for Potential Lithium–Air Battery Electrode
Source: Adv Sci (Weinh). 2017 May 19;4(9):1600453. doi: 10.1002/advs.201600453 (PMC5604394; doi:10.1002/advs.201600453)
Supplement: Supplementary file 1 — Supplementary [file ADVS-4-na-s001.pdf]

Copyright WILEY-VCH Verlag GmbH & Co. KGaA, 69469 Weinheim, Germany, 2017.

Supporting Information

for *Adv. Sci.,* DOI: 10.1002/advs.201600453

Oxygen-Rich Lithium Oxide Phases Formed at High Pressure for Potential Lithium–Air Battery Electrode

Wenge Yang, Duck Young Kim,* Liuxiang Yang, Nana Li, Lingyun Tang, Khalil Amine, and Ho-Kwang Mao*

Supporting Information for:

Oxygen-Rich Lithium Oxide Phases Formed at High Pressure for Potential Lithium-Air Battery Electrode

Wenge Yang*, Duck Young Kim*, Liuxiang Yang, Nana Li, Lingyun Tang, Khalil Amine, and Ho-kwang Mao

W, Yang, D. Y. Kim, L. Yang, N. Li, L. Tang, H. K. Mao
Center for High Pressure Science and Technology Advanced Research (HPSTAR), Shanghai 201203, China
* Email: yangwg@hpstar.ac.cn, duckyoung.kim@hpstar.ac.cn

W. Yang, L. Yang, L. Tang, H.K. Mao
High Pressure Synergetic Consortium (HPSynC), Geophysical Laboratory, Carnegie Institution of Washington, 9700 S Cass Avenue, Argonne, IL 60439, USA

K. Amine
Chemical Science & Engineering Division, Argonne National Laboratory, 9700 S Cass Avenue, Argonne, IL 60439, USA

Figure S1. Photograph of the diamond anvil surface after laser heating at 14.4 GPa.

Figure S2. XRD profiles of mixture of Li_2O_2 and oxygen under pressures near 15 GPa and 50 GPa at room temperature.

Figure S3. The DFT calculation of total energy of LiO_2 phase as a function of pressure.

Figure S4. The phonon dispersion curves of four lithium oxides at 48 GPa.

Figure S5. Reitveld refinement of XRD profile collected at 14.4 GPa after laser heating.

Table S1. The crystal phase, atomic position and the lattice parameters at 14.4 GPa determined from Reitveld refinement on the XRD data.

Table S2. The crystal structure, atomic positions and lattice parameters at 48 GPa from Reitveld refinement of XRD data.

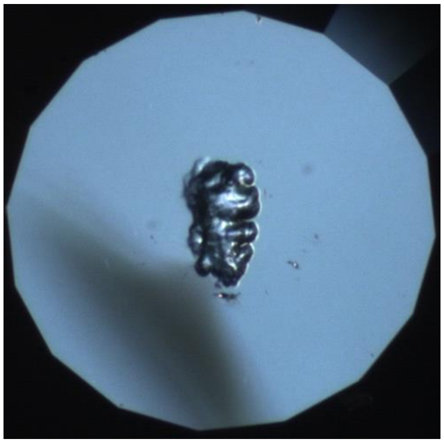

Figure S1. Photograph of the diamond anvil surface after laser heating at 14.4 GPa. Due to high power laser heating, partial carbon evaporated from anvil surface and participated in the redox reaction with Li_2O_2 to form Li_4CO_4 high-pressure phase. The black damaged region contributes to the broad diffraction peak near 8 degrees in the XRD patterns in Fig. 3 and Fig. S5.

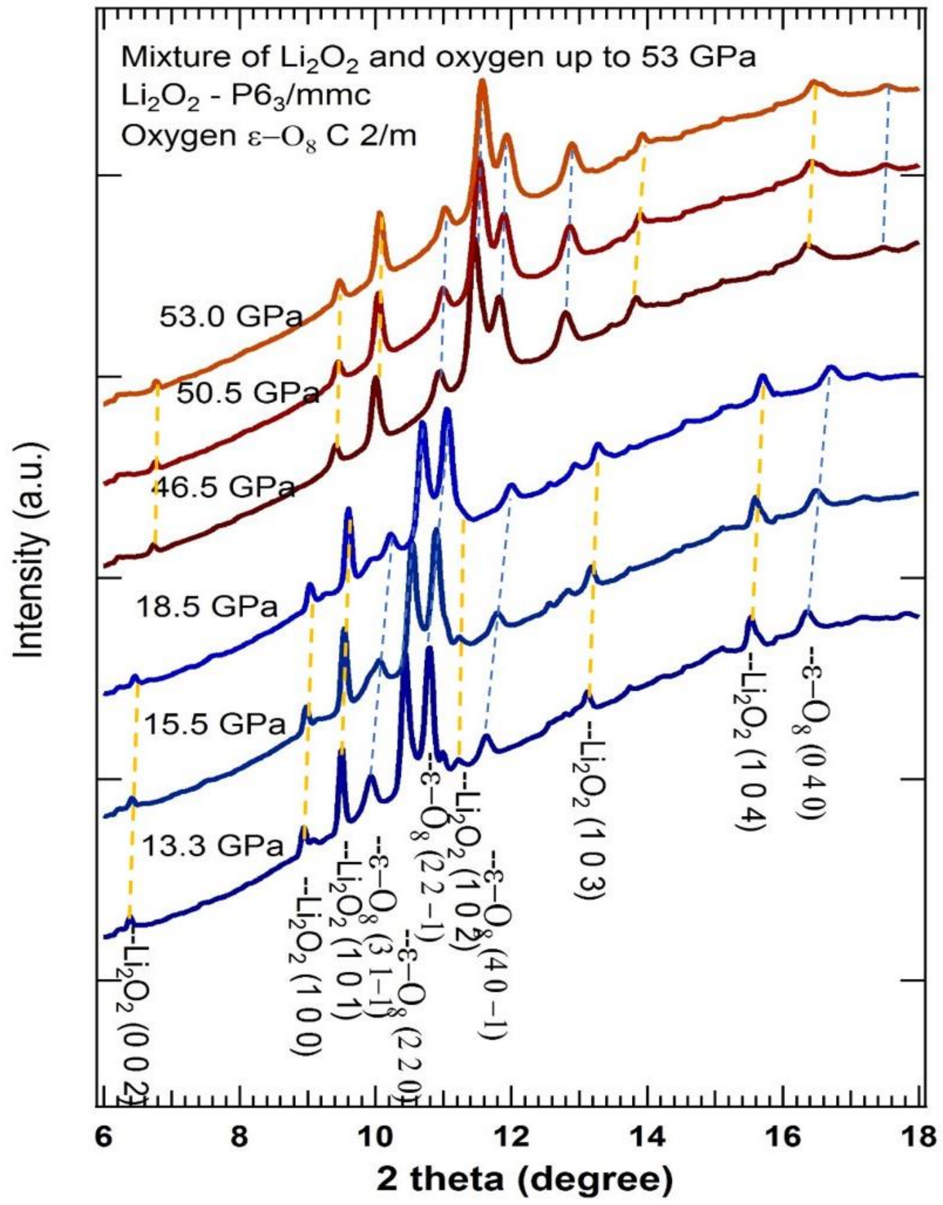

Figure S2. XRD profiles of mixture of Li_2O_2 and oxygen under pressures near 15 GPa and 50 GPa at room temperature. As labeled below the XRD profiles, all major diffraction peaks can be well indexed by Li_2O_2 ambient phase ($P6_3/mmc$) and ε-O_8 phase (C2/m) up to 53 GPa. The dashed lines in yellow and blue indicate the diffractions from Li_2O_2 and ε-O_8 phase, respectively.

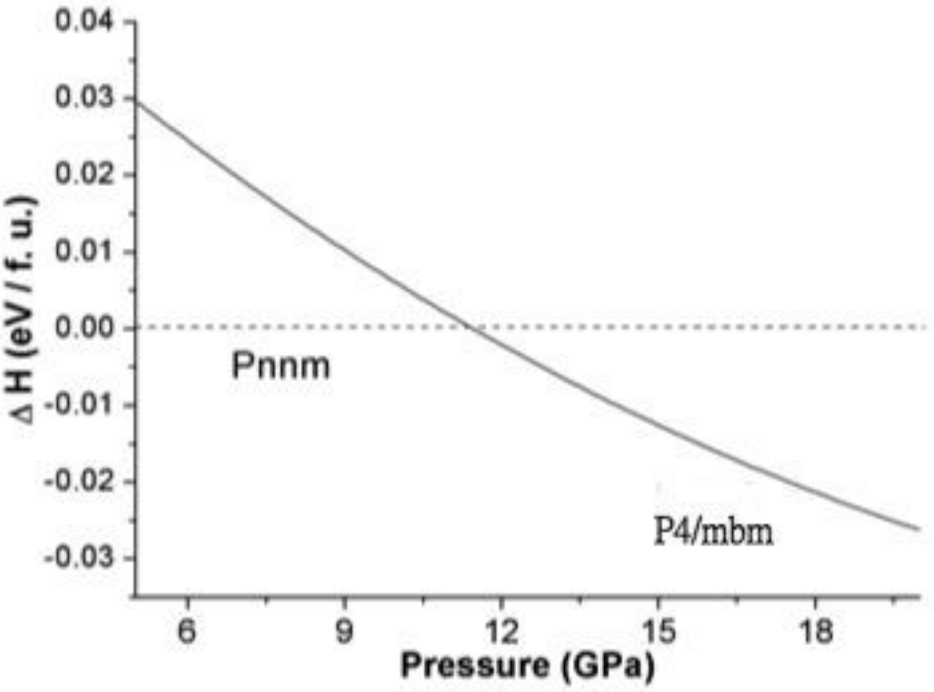

Figure S3. The DFT calculation of total energy of LiO_2 phase as a function of pressure. The ground state of LiO_2 changes from Pnnm to P4/mbm near 12 GPa. The dashed zero line is taken as reference for Pnnm phase, while the solid curve is the energy difference between P4/mbm and Pnnm phase.

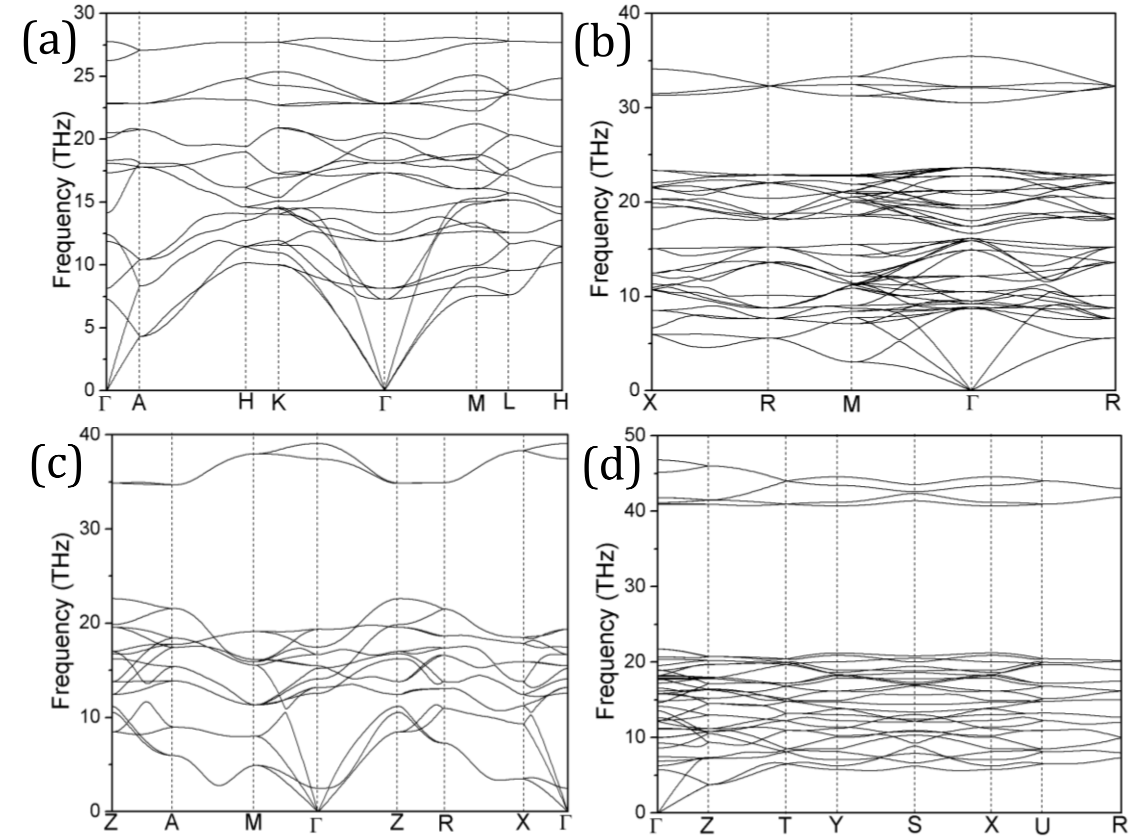

Figure S4. The phonon dispersion curves of four lithium oxides at 48 GPa. (a)-(d) are the phonon dispersion for Li_2O_2, Li_2O_3, LiO_2, and LiO_4 at 48 GPa, respectively. From these phonon dispersion results, one can see that all four components are thermodynamically stable at 48 GPa.

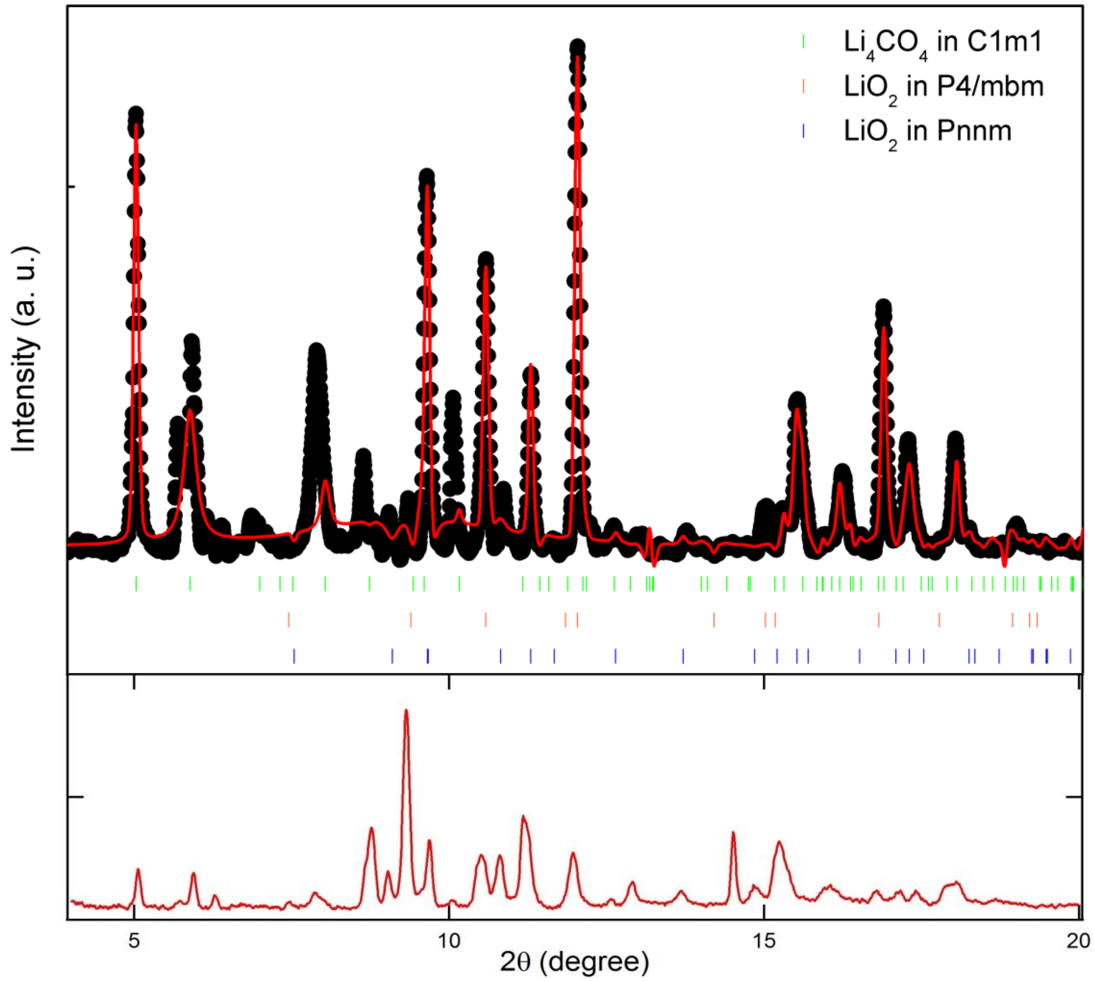

Figure S5. Reitveld refinement of XRD profile collected at 14.4 GPa after laser heating at the laser heating center and off-center locations. In the center location, the structure mainly consists of Li_4CO_4 (36.91%) and two types of LiO_2 forms: the ambient Pnnm (23.72%) and high pressure P4/mbm (39.37%) phase. For comparison, the off-center (30 microns away from the laser spot center) location only developed small amount of Li_4CO_4 comparing to the top XRD profile with not well formed LiO_2 P4/mbm and Pnnm phases (broad and low intensity peaks), which indicates the high temperature is important to synthesize the LiO_2 phases.

Table S1. The crystal phase, atomic position and the lattice parameters at 14.4 GPa determined from Reitveld refinement on the XRD data.

Phase	Lattice parameters	Atomic positions
Li_4CO_4 (C1m1, Z=2)	a=6.5545(15)Å, b=6.2389(19)Å, c=3.8813(9)Å, ß=95.458(29)º V=158.001(49)Å^3	C1 2a (0.00348 0 0.4987)<br>O1 4b (0.00048 0.81681 0.69332)<br>O2 2a (0.81207 0 0.24347)<br>O3 2a (0.18915 0 0.35486)<br>Li1 4b (0.31427 0.21696 0.00993)<br>Li2 4b (0.20302 0.70794 0.47902)
LiO_2 (Pnnm, Z=2)	a=3.9338(28)Å, b=4.7425(6)Å, c=2.9665(7)Å, V=55.342(21)Å^3	Li 2a (0 0 0)<br>O 4g (0.133 0.4156 0)
LiO_2 (P4/mbm, Z=2)	a=b=4.3326(6)Å, c=2.4370(3)Å, V=45.741(11)Å^3	Li 2b (-1.5 -2.5 0.5)<br>O 4g (-1.89021 -1.60979 -1)

Table S2. The crystal structure, atomic positions and lattice parameters at 48 GPa from Reitveld refinement of XRD data.

Phase	Lattice parameters	Atomic positions
LiO_4 (Ibam, Z=4)	a=4.3818(21)Å, b=4.3944(9)Å, c=7.0155(126)Å V=135.091(211)Å^3	Li 4b (0 0.5 0.25) O 16k (0.1753 1.1692 1.0923)
LiO_2 (P4/mbm, Z=2)	a=b=4.0365(14)Å, c=2.2021(35)Å, V=35.881(62)Å^3	Li1 2a (0 0 0) Li2 8i (0.5 0.5 0) O1 4h (0.1102 0.6103 0.5) O2 4h (0.8864 0.3862 0.5) O3 8j (0.3862 0.1102 0.5) O4 8j (0.6103 0.8864 0.5)
Li_2O_3 (Im-3m, Z=2)	a=b=c=5.1866(13)Å, V=139.524(62)Å^3	Li 8c (-0.75 0.25 -0.25) O 12e (-0.5 0.5 -0.1385)
Li_2O_2 (P6$_3$/mmc, Z=2)	a=b=2.9628(12) Å, c=6.7850(66) Å, V=51.583(61Å^3	Li1 2c (0.3333 0.6667 0.25) Li2 2a (0 0 0) O 4f (0.3333 0.6667 0.6320)
O_2 (C2/m, Z=2)	a=7.4032(57)Å, b=5.0244(22)Å, c=3.5155(14)Å, ß=116.517º V=117.012(60) Å^3	O1 8j (0.0296 0.2507 0.1792) O2 4i (0.2437 0 0.1830) O3 4i (0.1891 0 -0.1813)
